# Effects of traditional Chinese herbal medicine in patients with diabetic kidney disease: study protocol for a randomized controlled trial

**DOI:** 10.1186/s13063-018-2749-6

**Published:** 2018-07-17

**Authors:** Mengdi Wang, Zhen Wang, Jingwei Zhou, Weiwei Sun, Ying Wang, Mei Han, Hanwen Yang, Wei Jing Liu, Yaoxian Wang

**Affiliations:** 1 Department of Nephrology, Dongzhimen Hospital Affiliated to Beijing University of Chinese Medicine, 5 Haiyuncang, Dongcheng District, Beijing, 100700 China; 2 Institute for Nephrology Research of Beijing University of ChineseMedicine, 5 Haiyuncang, Dongcheng District, Beijing, 100700 China; 3 Key Laboratory of Chinese Internal Medicine of Ministry of Education and Beijing, Beijing University of Chinese Medicine, Dongzhimen Hospital Affiliated to Beijing University of Chinese Medicine, 5 Haiyuncang, Dongcheng District, Beijing, 100700 China; 4 Center for Evidence-based Medicine of Beijing University of Chinese Medicine, 11 Beisanhuandonglu, Chaoyang District, Beijing, 1000029 China

**Keywords:** Chinese herbal medicine, Diabetic kidney disease, Randomized controlled trial

## Abstract

**Background:**

Diabetic kidney disease (DKD) is a major microvascular complication of diabetes mellitus and the primary cause of end-stage renal disease. Existing therapies for DKD are not sufficiently effective. We report the protocol of a pragmatic randomized controlled trial of the use of traditional Chinese herbal medicine to treat patients with DKD.

**Methods/design:**

This will be a multicenter randomized controlled trial. A total of 266 patients with DKD (106 with early stage, 80 with middle-stage, and 80 with advanced-stage disease) with an estimated glomerular filtration rate (eGFR) ≥ 30 mL/min/1.73m^2^will be included. Participants with DKD of each stage will be randomly allocated at a 1:1 ratio to either the experimental group, which will receive *Xiaozhen* formula and basic treatment, or the control group, which will receive basic treatment only. The study duration will be 24 weeks. The primary outcome will be urinary microalbumin excretion rate for early stage DKD, 24-h urinary protein for middle-stage DKD, and eGFR for advanced-stage DKD. Adverse events will also be evaluated. Data for all outcome indicators will be collected at baseline and weeks 4, 12, and 24.

**Discussion:**

This study will provide evidence of the effectiveness and safety of traditional Chinese herbal medicine in treating patients with DKD.

**Trial registration:**

Chinese Clinical Trials Registry: ChiCTR-IOR-16010072. Registered on 2 December 2016.

**Electronic supplementary material:**

The online version of this article (10.1186/s13063-018-2749-6) contains supplementary material, which is available to authorized users.

## Background

Diabetic kidney disease (DKD) is a major microvascular complication of diabetes mellitus (DM).With the prevalence of diabetes increasing, the incidence of DKD is growing rapidly, not only in the Western world but also in some developing countries. Thus, the number of patients with chronic kidney disease (CKD) related to DM in China, recently estimated to be 24.3 million, has exceeded the number of patients with CKD related to glomerulonephritis [1]. DKD is associated with an extremely high risk of progression to end-stage renal disease (ESRD) and cardiovascular morbidity and mortality [2, 3], emphasizing the importance of early prevention and treatment. However, few effective therapies are currently available. In addition to glycemic control, renin-angiotensin-aldosterone system (RAAS) inhibition has remained the mainstay of the treatment for DKD for a long time. Many experimental studies and clinical trials have demonstrated that RAAS inhibition with angiotensin-converting-enzyme inhibitors (ACEIs) or angiotensin receptor blockers (ARBs) can reduce microalbuminuria and improve renal function, slowing the progression to ESRD [4–6]. However, many adverse effects of ACEIs and ARBs, such as hyperkalemia [7], acute kidney injury [8], rhinitis, persistent cough, and angioedema [9, 10], preclude the continuous use of this therapy in many cases. Moreover, patients with a low estimated glomerular filtration rate (eGFR) usually cannot receive RAAS inhibitors. Although new therapies have been tried in the recent years, few have been proven to be both effective and safe [11–13].

In China, many patients with CKD resort to traditional Chinese herbal medicine (TCHM).Many in vitro and animal studies have demonstrated the biological activity and therapeutic effects of TCHM [14, 15]. However, substantial variations in TCHM individual treatment patterns and the need for frequent adjustments make it difficult to design a strict modern controlled trial for traditional Chinese therapy. Hence, high-quality randomized controlled trials, and especially well-designed clinical trials, of traditional Chinese medicine (TCM) are rare.

The *zhenjia* theory is first mentioned in the *Inner Canon of the Yellow Emperor* (*Huang di Nei jing*). *Zhenjia* refers to a complex of phlegm, stasis, heat, and damp that mainly forms and exists in collaterals. In CKD, *zhenjia* forms in the collaterals of the kidney and exacerbates kidney injury. TCHM can help dissolve *zhenjia*. Our previous studies have shown that a formula based on the *zhenjia* theory can decrease urinary albumin and serum creatinine (Scr) levels and suppress interstitial expansion and glomerulosclerosis in rats with DKD. These therapeutic effects might be related, at least partially, to the inhibition of inflammatory response and extracellular matrix accumulation mediated by the TNF-α/NF-κBp65 signaling pathway [16]. According to the *zhenjia* theory, the composition of *zhenjia* is different in different stages of DKD. Thus, *zhenjia* is mainly composed of heat, stasis, and turbidity toxin in the early, middle-, and advanced-DKD stages, respectively. Therefore, it is necessary to dissipate the heat, remove the blood stasis, and dislodge the turbid toxin. Accordingly, we prescribe different formulas to patients with DKD of different stages. The *Tourexiaozhen* formula is prescribed to patients with early stage DKD to dissipate heat; the *Huoxuexiaozhen* formula is utilized for middle-stage DKD to remove blood stasis; while the *Xiezhuoxiaozhen* formula is employed for advanced-stage DKD to dislodge the turbid toxin. The proposed study will be designed as a randomized controlled trial that evaluates DKD treatment according to the disease stages. In order to recapitulate the real-world practice of TCM, it will be possible to adjust each formula individually according to the patient’s signs and symptoms.

## Methods/design

### Study design

**Fig. 1:**
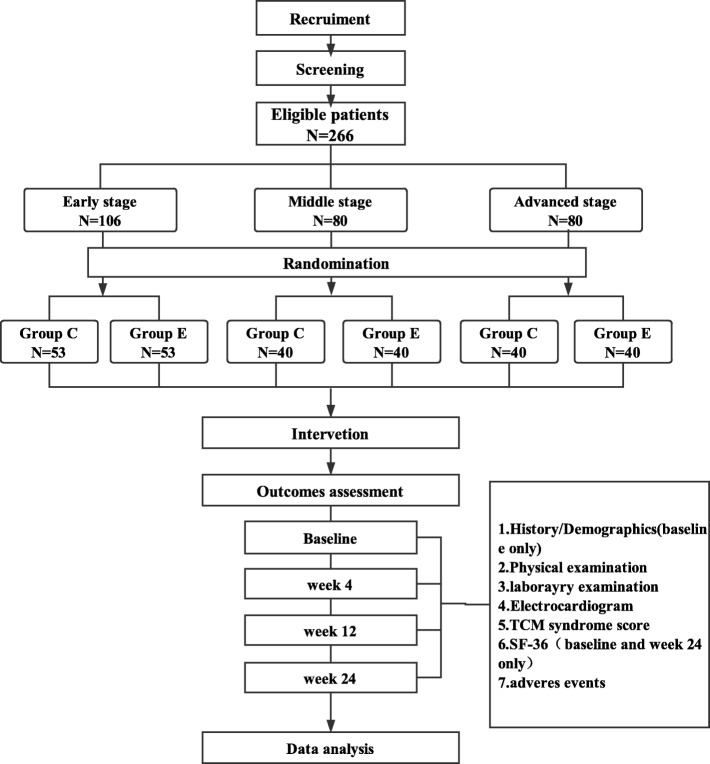
Study flowchart. Group C: control group. Group E: experimental group

The proposed study is a prospective, multicenter, assessor- and analyst-blinded, pragmatic randomized controlled trial. A total of 266 participants will be recruited at the following six tertiary A hospitals of TCM in Beijing: Dongzhimen Hospital Affiliated to Beijing University of Chinese Medicine, Guang’anmen Hospital, China Academy of Chinese Medical Sciences, Beijing Hospital of Traditional Chinese Medicine, Wangjing Hospital, China Academy of Chinese Medical Sciences, Xiyuan Hospital, China Academy of Chinese Medical Sciences, Beijing Hospital of Integrated Traditional Chinese and Western Medicine, and Fangshan Traditional Medical Hospital of Beijing. Eligible patients who agree to participate will be randomly allocated at a 1:1 ratio to either the experimental group or the control group according to disease stage. After randomization, patients in the experimental group will receive basic treatment and TCHM, whereas patients in the control group will receive basic treatment only. The treatment will continue for 24weeks in both groups. Four visits will be scheduled for each patient: weeks 0 (baseline), 4, 12, and 24. The study flowchart is shown in Fig. 1. The Standard Protocol Items: Recommendations for Interventional Trials (SPIRIT) Checklist is presented in Additional file 1.

### Participants

#### Diagnostic criteria

The diagnostic criteria of this trial will be set based on the National Kidney Foundation Kidney Disease Outcomes Quality Initiative (NKF-KDOQI) guidelines [17], the Kidney Disease Improving Global Outcomes (KDIGO) 2012 Clinical Practice Guideline for the Evaluation and Management of Chronic Kidney Disease [18], and the Consensus for Prevention and Treatment of Diabetic Kidney Disease 2014 of Chinese Diabetes Society [19]. For a diagnosis of DKD, patients with DM will have to have at least one of the following conditions:

1. Albuminuria (for microalbuminuria, out of three tests, two repeated tests performed within 3–6 months will need to produce abnormal results)
2. Diabetic retinopathy with CKD
3. DKD confirmed by renal biopsy

#### Stage differentiation

1. Early stage DKD: urinary microalbumin excretion rate (UAER) of 30–300 mg/24 h or urinary albumin-to-creatinine ratio (ACR) of 30–300 mg/g (mg/mmol)
2. Middle-stage DKD: UAER > 300 mg/24 h or ACR > 300 mg/g (mg/mmol), or 24-h urinary protein (24-h UP) > 0.5 g and eGFR ≥ 60 mL/min/1.73m^2^
3. Advanced-stage DKD: eGFR of 30–60 mL/min/1.73m^2^

#### Inclusion criteria

1. Presence of DKD according to the above diagnostic criteria
2. Age between 25 and 75 years
3. eGFR ≥ 30 mL/min/1.73m^2^ without dialysis
4. For those with hypertension, controlled blood pressure ≤ 140/90 mmHg; for the elderly, systolic blood pressure (SBP) ≤ 150 mmHg
5. Glycated hemoglobin (HbA1c) level ≤ 7%; for those with repeated hypoglycemia, severe microvascular or macrovascular complications, other severe comorbidities, or difficulties in achieving the standard requirements in spite of adequate therapy, HbA1c ≤ 8%
6. Signed informed consent

#### Exclusion criteria

1. Severe infection, anemia, electrolyte imbalance, or acute complications of DM in the previous 4 weeks
2. Severe cardiac, cerebral, hepatic, or hemorrhagic diseases, including cerebral infarction, cerebral hemorrhage, transient ischemic attack, myocardial infarction, unstable angina, heart failure, and hepatic inadequacy with aspartate transaminase (AST) or alanine aminotransferase (ALT) levels more than twice the normal upper limit
3. Use of corticosteroids or immunosuppressants in the previous 3 months
4. Oliguria, anuria, severe edema, massive pleural or peritoneal effusion
5. Renal transplantation
6. Mental disorders
7. Pregnancy or lactation
8. Allergy to the trial drugs
9. Participation in other clinical studies
10. Incomplete understanding of the study, refusal to participate, or lack of signed informed consent

#### Handling of withdrawal and dropout

Participants will be able to withdraw from the study for any reason at any time, and the reason will be recorded faithfully in the case report form (CRF). Investigators can also remove participants from the study to ensure their safety or maintain the quality of the trial. Participants with any of the following conditions may be removed from the study:

1. Occurrence of diseases or conditions mentioned in the exclusion criteria during the course of the study
2. Occurrence of severe complications and/or general health deterioration
3. Occurrence of serious adverse events (SAEs)
4. Rapidly increasing Scr level (more than 50% of baseline) or ESRD
5. Violation of the study protocol
6. Voluntary withdrawal from the trial or loss to follow-up

### Randomization and blinding

The random sequence will be generated by the Center for Evidence-Based Medicine of Beijing University of Chinese Medicine using Statistical Analysis System (SAS version 9.2), and randomization will be stratified according to study center and DKD stage. Eligible patients with DKD of each stage will be randomized to the experimental group or the control group at a 1:1 ratio. The group numbers will be provided in envelopes made from carbonless paper. At each center, the envelopes will be kept by a study administrator who will not directly participate in the recruitment or follow-up of any participant, and the group numbers will be subsequently disclosed. The administrator will open one envelope and provide the participant with their group number on the day of inclusion.

Different people will serve as investigators, therapists, and assessors. Assessors of primary and secondary endpoints and data analysts will be blinded to the treatment allocation throughout the course of the study, while patients, investigators, and therapists administering the treatment will not be blinded.

### Intervention

Participants with DKD of each stage will receive basic treatment and TCM or basic treatment only for 24 consecutive weeks according to the random assignment.

#### Control group

Participants in the control group will receive basic treatment for DKD based on the recommendations of Treatment of Diabetic Kidney Disease 2014 by the Chinese Diabetes Society, including blood glucose control (HbA1c will be maintained below 8% throughout the trial), blood pressure control (participants with hypertension will receive antihypertensive drugs to maintain their blood pressure ≤ 130/80 mmHg, except for individuals aged 60 years or older, for whom the systolic pressure threshold will be ≤ 150 mmHg.

#### Experimental group

In addition to the above-mentioned basic treatment, participants in the experimental group will receive an oral traditional Chinese formula based on the *Zhenjia* theory, which is different for different stages of DKD. The *Tourexiaozhen* formula for participants with early stage DKD will consist of *Radix scutellariae* (*Huang Qin*), *Fructus forsythiae* (*Lian Qiao*), *Fructus arctii* (*Niu Bang Zi*), and *Radix scrophulariae* (*Xuan Shen*); the *Huoxuexiaozhen* formula for those with middle-stage DKD will include *Fructus arctii* (*Niu Bang Zi*), *Abelmoschus corolla* (*Huang Shu Kui Hua*), *Sargassum* (*Hai Zao*), *Hirudo* (*Shui Zhi*), and *Radix astragali* (*Huang Qi*); and the *Xiezhuoxiaozhen* formula for end-stage DKD will include *Radix astragali* (*Huang Qi*), *Cortex eucommiae* (*Du Zhong*), *Eupolyphaga seu steleophaga* (*Tu Bie Chong*), *Sargassum* (*Hai Zao*), *Radix et rhizoma rhe* (*Da Huang*), and *Rhizoma smilacis glabrae* (*Tu Fu Ling*). The ingredients in each formula can be adjusted individually at every visit according to patient’s signs and symptoms, and the dose of each ingredient in the prescription will be decided by the physician. The standard adjustments will be as follows: for patients with dry mouth or thirst, *Radix puerariae* (*Ge Gen*) will be added to the formula; for patients with bitter taste, *Radix bupleuri* (*Chai Hu*), *Radix scutellariae* (*Huang Qin*), and *Fructus aurantii immaturus* (*Zhi Shi*) will be added to the formula; for patients with low back pain, *Radix cyathulae* (*Chuan Niu Xi*), *Dipsaci radix* (*Xu Duan*), and *Rhizoma cibotii* (*Gou Ji*) will be added to the formula; for patients with edema, *Polyporus* (*Zhu Ling*), *Poria* (*Fu Ling*), *Rhizoma alismatis* (*Ze Xie*), *Pericarpium arecae* (*Da Fu Pi*), and *Exocarpium benincasae* (*Dong Gua Pi*) will be added to the formula; for patients with abdominal distension, *Radix aucklandiae* (*Mu Xiang*), *Fructus amomi* (*Sha Ren*), *Citrus medica* (*Xiang Yuan*), and *Fructus citri sarcodactylis* (*Fo Shou*) will be added to the formula; and for patients with itching skin, *Fructus kochiae* (*Di Fu Zi*) will be added to the formula. All formulae will be prescribed by a physician licensed in Chinese medicine and with at least 8 years of clinical experience. The formulae will be prepared in the form of granules by Beijing Tcmages Pharmaceutical Co., Ltd. (Beijing, China), a company that has passed the Chinese Good Manufacturing Practice for Pharmaceutical Products certification. A single-day supply will be provided in two doses (pouches) to be taken orally one at a time (twice a day).

### Outcomes

#### Primary outcomes

For early stage DKD, the primary outcome will be UAER. For middle-and advanced-stage DKD, the primary outcomes will be 24-h UP and eGFR, respectively.

#### Secondary outcomes

1. Physicochemical indicators: HbA1c, eGFR, Scr for early and middle-stage DKD and HbA1c, Scr, and blood urea nitrogen (BUN) for advanced-stage DKD
2. TCM syndrome integral score, which will be determined using the TCM Syndrome Integral Scale
3. Patient quality of life evaluated using Short Form 36-item Health Survey (SF-36)
4. Microinflammation indicators: C-reactive protein (CRP), interleukin-6 (IL-6), and tumor necrosis factor α (TNF-α) levels

#### Safety outcomes

Complete blood count (CBC), AST, ALT, alkaline phosphatase (ALP), glutamyl transpeptidase (GGT), and total bilirubin (TBIL) tests and electrocardiography will be performed at baseline and at every study visit. The proportion of patients demonstrating adverse drug reactions (ADRs) in the experimental group will be compared with that in the control group. ADRs will be defined as undesirable, unexpected symptoms, signs, or diseases that occur during the clinical trial and can be caused by the treatment.

#### Laboratory tests

Routine laboratory tests (CBC and general urine analysis, Scr, BUN, AST, ALT, ALP, GGT, UAER, 24-h UP, and HbA1c) will be performed by the clinical laboratory of each center. For CRP, IL-6, and TNF-a level determination, an additional 3–5 mL of venous blood will be collected. The serum will be separated by centrifugation for 15 min at 3000×g, aliquoted, labeled concisely, and stored at − 80 °C until the final analysis. Repeated freeze-thaw cycles will be avoided. The inflammation indicators will be measured at the Research Center, Dongzhimen Hospital Affiliated to Beijing University of Chinese Medicine using specific ELISA kits.

### Adverse event (AE) reporting

AEs will be defined as undesirable, unexpected symptoms, signs, or diseases regardless of whether related to the treatment. If any AE happens, the investigators will take proper measures (dose adjustment, drug withdrawal, or symptomatic treatment) to ensure safety of the participant and perform continuous follow-up until the patient’s condition returns to normal. The relationship between the AEs and the experimental treatment will be further evaluated. All AEs and their characteristics, including the manifestation, onset date and time, duration, cause, relationship with the experimental treatment, specific therapy, and outcome, will be recorded.

In case of a severe AE (SAE) that can result in death, disability, hospitalization, or prolonged hospitalization, in addition to the above-mentioned measures, the chief investigator will be notified immediately and a written report will be submitted to the Research Ethics Committee within 24 h.

### Data collection

**Fig. 2:**
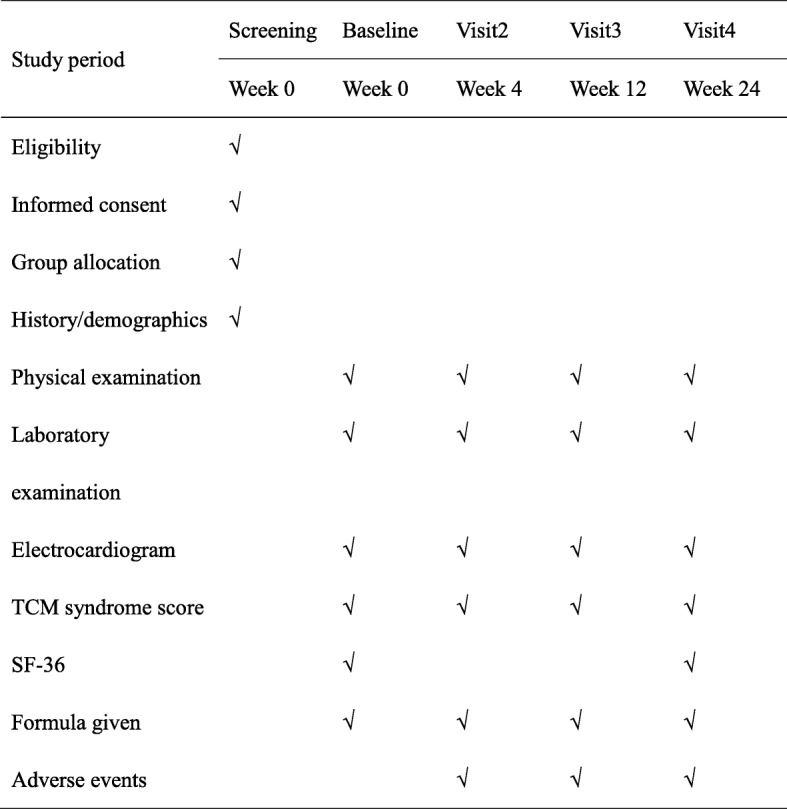
Standard Protocol Items: Recommendations for Interventional Trials (SPIRIT) Figure. Laboratory examination includes complete blood count (CBC) and general urine analysis, potassium, calcium, phosphates, fasting blood glucose, liver function tests (AST, ALT, ALP, GGT, TBIL), renal; function (BUN, Scr), and serum lipids (cholesterol, triglycerides, and low-density lipoprotein levels), UAER for patients with early stage DKD, and 24-h UP and HbA1c for weeks 0, 12, and 24

A standardized CRF designed for this study will be used to collect relevant data. The participants will be evaluated at baseline and weeks 4, 12, and 24. Each visit will have to occur within 5 days from the scheduled time. Information about the participants, including demographics, medical history, and previous medications, will be collected at baseline. Data on symptoms and TCM syndrome and results of physical examination and laboratory tests will be collected at each visit; HbA1c will be measured at baseline and at weeks 12 and 24. SF-36 will be administered only at the first and last visits. The schedule of the trial can be found in Fig. 2.

### Quality assurance and data management

To ensure the quality of the study and its adherence to the protocol, physicians responsible for prescribing the experimental formula will be trained beforehand, whereas all investigators involved in the study will be trained in recruiting participants and will be provided a written protocol and a standardized record form.

All information from CRFs will be carefully recorded. Clinically unacceptable data will require investigation and explanation by the researcher. All errors will need to be corrected by crossing out, with the researcher’s signature and date. Withdrawals or/and missed follow-up visits will need to be explained in CRFs. After one participant completes the study at each center, the trial monitor will visit the site. The monitor will review the CRFs, check the inclusion, exclusion, and withdraw criteria, and compare the data in the CRFs to those in the source medical records. Any inadequacies or errors will be reviewed by the local investigator and coordinator.

Data from CRFs will ultimately be input into an electronic database (Epidata 3.1) designed for the trial. Data will be entered twice (independently by different persons). After the independent double entry, data will be checked by the database software. All non-identical items will be checked against the source medical records and corrected. Additionally, 10 CRFs will be randomly selected and compared manually with corresponding database entries. All study-related data and documentation will be retained at the Institute for Nephrology Research of Beijing University of Chinese Medicine. All personal information of potential and enrolled participants will be maintained to protect confidentiality before, during, and after the trial.

### Sample size

To estimate the group size, a small-scale study was conducted to measure the effects of TCHM in 90 patients, with 30 patients with DKD of each stage. Half of the patients regularly received herbal therapy, while the other half received basic treatment only. After 24 weeks, UAER in the control and experimental groups (early stage DKD) was70 ± 51 mg and 44 ± 27 mg, respectively, 24-h UP in the control and experimental groups (middle-stage DKD) was 3.25 ± 1.09 and 2.50 ± 0.95 g, respectively, and eGFR in the control and experimental groups (advanced-stage DKD) was 33.4 ± 9.1 and 40.5 ± 10.2 mL/min/1.73m^2^, respectively. Using early stage DKD as an example, the following formula was employed to calculate the sample size that would allow obtaining a significantly lower UAER in the experiment group after 24 weeks of treatment:

$$ n={\left({\mu}_{\upalpha}+{\mu}_{\upbeta}\right)}^2\times \left(1+1/\mathrm{k}\right)\times {\sigma}^2/{\left({\mu}_2-{\mu}_1\right)}^2, $$

where *σ*^2^is overall variance, which was estimated as sample variance s^2^:

$$ {s}^2=\left({s_1}^2+\mathrm{k}{s_2}^2\right)/\left(1+\mathrm{k}\right). $$

The proportion of cases between the experimental and control groups was set to 1:1(k = 1). The study was designed to have a power of approximately 90% and a one-side level of significance of 0.05 (*α* = 0.05, *β* = 0.1). *μ*_1_, *μ*_2_, *s*1, and *s*_2_ are mean and standard deviations in the control and experimental groups. This resulted in *n* = (1.64 + 1.28)^2^ × (51^2^ + 27^2^/(70 − 44)^2^ = 42.

Thus, after assuming a dropout rate of 20%, the sample size for the early stage subgroup in each group was determined to be 53. Similar calculations resulted in a sample size of 40 patients for the middle-stage and advanced-stage subgroups in each group. This produced the final sample size of 266 patients.

### Statistical analysis

Statistical analysis will be conducted at the Center for Evidence-Based Medicine of Beijing University of Chinese Medicine using Statistic Package for Social Science (SPSS, version 22.0, Beijing University of Chinese Medicine). Intention-to-treat and per protocol sets will be used to analyze the efficacy of TCHM. A safety analysis set will be used to evaluate the safety of the trial. Missing outcome variables will be estimated using the principle of the last observation carried forward, with the data from the last study follow-up used as the final results.

The independent-samples *t* test or Mann-Whitney test will be used for comparison of the trial primary outcomes between the two groups, including UAER in the early stage subgroup, 24-h UP in the middle-stage subgroup, and eGFR in the advanced-stage subgroup, which are all continuous variables with four measurements. Moreover, repeated measures analysis of variance will be attempted to further test the time effect and treatment cross-effect. With regard to the secondary outcomes, the independent-samples *t* test or Mann-Whitney test will be used to analyze continuous variables, and the chi-squared test will be used to analyze classified variables. With regard to safety outcomes, the incidence of AEs will be determined and a comparison between the two groups will be attempted. A stratified analysis of data according to DKD stage or center will be attempted in order to decrease bias. A *P* value < 0.05 will be considered to indicate a statistically significant difference.

## Discussion

DKD is a worldwide health problem. It has been estimated that approximately 25–40% of patients with DM will develop DKD [20]. Compared to non-diabetic CKD, DKD progresses more rapidly to ESRD [21, 22]. Moreover, owing to other concomitant complications of DM, such as diabetic retinopathy and diabetic foot, patients with DKD are often in poor health and have low quality of life. TCHM has been long used to treat complications of DM, including DKD, and has a great ability to improve patients’ quality of life. As we know, TCM is an individual treatment based on the theory of syndrome differentiation. A prescription usually consists of several well-chosen herbs is one of the inherent characteristics of TCM, while sometimes it needs adjustments owing to its principle of individual treatment. Chinese herbal medicine has been proved as an effective way to treat DKD by many researchers. However, most of the reported formula was unified prescription or a single herb under ideal circumstances. Here, we try to explore an effective research method to show the effect of *xiaozhen* therapy in DKD patients and reflect the characteristic of TCM as our real daily clinical practice. So, our study is designed as a randomized controlled trial in which all participants will be randomly assigned either to the control group or the experimental group, with very similar controlled conditions in the two groups. Simultaneously, it is a pragmatic trial in which the ingredients of the formula can be adjusted by physicians according to patient’s symptoms and signs. This is more in accordance with the above characteristic of TCM. Recently, a prospective research study from Taiwan has shown that patients with CKD who used prescription Chinese medicine had a significantly (60%) reduced ESRD risk [23]. A similar study for DKD is lacking. Our study has the potential to contribute to the development of an effective DKD treatment.

Some limitations of our study should be acknowledged. First, there may be differences between the six centers included in the trial in terms of experience in using TCHM for DKD. Therefore, a strict training and supervision system will be implemented to ensure that the trial is as homogeneous and consistent as possible. Second, because participants will not be blinded to their allocation, assignment to the unwanted group may result in poor compliance. We will describe the trial to the participants before they enter the study and perform telephone follow-up to avoid this potential problem. Last, the sample size of this study was based on our preliminary study while taking herbal therapy including the adjustments as an interfering group, the samples of our study may be different if each adjustment be considered separately. As a trial in TCM research, it needs more improvements in the future.

## Trial status

We are currently recruiting participants.

## Additional file

Additional file 1: Standard Protocol Items: Recommendations for Interventional Trials (SPIRIT) Checklist. (DOC 135 kb)

## Abbreviations

24-h UP: 24-h urinary protein
ACEI: Angiotensin converting enzyme inhibitor
ACR: Albumin-to-creatinine ratio
ADR: Adverse drug reaction
AE: Adverse event
ALP: Alkaline phosphatase
ALT: Alanine aminotransferase
ARB: Angiotensin receptor blocker
AST: Aspartate transaminase
BUN: Blood urea nitrogen
CBC: Complete blood count
CKD: Chronic kidney disease
CRF: Case report form
CRP: C-reactive protein
DKD: Diabetic kidney disease
DM: Diabetes mellitus
eGFR: Estimated glomerular filtration rate
ESRD: End-stage renal disease
GGT: Glutamyl transpeptidase
HbA1c: Glycated hemoglobin
IL-6: Interleukin-6
KDIGO: Kidney Disease Improving Global Outcomes
NKF-KDOQI: National Kidney Foundation Kidney Disease Outcomes Quality Initiative
RAAS: Renin-angiotensin-aldosterone system
SAE: Severe adverse event
SAS: Statistical Analysis System
SBP: Systolic blood pressure
Scr: Serum creatinine
SF-36: Short Form 36-item Health Survey
SPSS: Statistic Package for Social Science
TBIL: Total bilirubin
TCHM: Traditional Chinese herbal medicine
TCM: Traditional Chinese medicine
TNF-α: Tumor necrosis factor α
UAER: Urinary microalbumin excretion rate
